# The Self-Adaptive Fuel Supply Mechanism in Micro DMFC Based on the Microvalve

**DOI:** 10.3390/mi10060353

**Published:** 2019-05-29

**Authors:** Zhenyu Yuan, Wenhui Chuai, Zhongming Guo, Zhaoyin Tu, Fanbo Kong

**Affiliations:** College of Information Science and Engineering, Northeastern University, Shenyang 110819, China; chuaiwenhuiyc@126.com (W.C.); guo_zhongming@126.com (Z.G.); tuzhaoyin@126.com (Z.T.); K1585817556@126.com (F.K.)

**Keywords:** direct methanol fuel cell, microvalve, self-adaptive, thermal control

## Abstract

To achieve a self-adaptive fuel supply mechanism for the micro direct methanol fuel cell (μDMFC), we designed and developed a thermal control microvalve channel structure, where we considered the relationship between the temperature characteristics, viscosity, and velocity of the methanol solution. Both the single channel model and three-dimensional cell model for the microvalve were established using the COMSOL Multiphysics program. The results demonstrated that in the microvalve channel, the viscosity of the solution decreased, and the flow rate at the microvalve outlet increased with the increasing temperature. Meanwhile, the geometry structure of the microvalve single channel was optimized, so that the effect of the control speed of the microvalve under temperature changes became more prominent. In the full-cell model analysis, a low-velocity methanol solution at the low current density can significantly inhibit methanol crossover. At the high current densities, an increase in the methanol solution flow rate was beneficial to an increase in the cell reaction output. The μDMFC was fabricated and the experiment was conducted, where the results showed that the power density of the self-adaptive cell reached a maximum value of 16.56 mW/cm^2^ in 2 M methanol solution, which was up to 7% better than conventional cell performance. The proposed microvalve structure can effectively improve the output power of the μDMFC during the whole reaction process, and it may improve the stability of the cell operation.

## 1. Introduction

The micro direct methanol fuel cell is a promising micro energy source with broad potential for application citing its advantages of high energy conversion efficiency, strong continuous power supply capability, being environmental-friendly, low temperature and fast start, high reliability, and easy integration [1,2,3]. Direct methanol fuel cells (DMFCs) can be classified into two types according to the supply mode of the fuel and oxidant, that is, active cells and passive cells [4]. Passive cells rely primarily on free diffusion to control the fuel and oxidant supply, and the cell output performance is relatively low. In active cells, the fuel and oxidant supply needs to be controlled by active auxiliary devices, such as pumps and valves, with particular flow rates and concentration, which is not conducive in portable applications of DMFC. Therefore, ascertaining how to combine the advantages of the two types of cells is of great significance to the practical application of DMFCs.

The liquid supply mechanism of the DMFC has been extensively studied, to improve the cell energy density and stability. Yang et al. [5] investigated the effects of different anode flow fields and parameters on the cell performance, where the results showed that the single-serpentine flow field exhibited a significantly higher performance than the parallel flow fields. Similarly, in their research, Deng et al. [6] reached the same conclusion. Adnan Ozden [7] applied Murray’s Law to design two different bio-inspired, leaf-shaped flow fields. The serpentine, the lung, and the two leaf-shaped flow fields were used to form seven different anode–cathode combinations. For comparison, when patterning the serpentine flow field, the peak power density was 824 W/m^2^.

Specifically, the working parameters have a significant and overlapping impact on the output performance of the μDMFC. These parameters include temperature, methanol solution concentration, flow rate, and the cell operating temperature. Given these factors, the internal temperature transport has been the focus of ongoing research [8,9]. A.A. Kulikovsky [10] reported a model that considered fragment heating due to reactions and cooling by the fuel flow in the anode channel and by water evaporation. Y. Wang [11] designed a spatial model of the DMFC, as well as a system model, including the air supply and the pump for the methanol supply. The results described the whole cell temperature distribution. In addition, the effect of the anode methanol flow rate on the performance of the μDMFC is complex, citing the need to control several key factors that affect cell performance, including reactant mass transfer, gas CO_2_ emission, and methanol crossover. On the one hand, an increase in the flow rate leads to an increase in the mass transfer rate, which can effectively overcome the mass transfer resistance of the anode and increase the bubble CO_2_ discharge velocity in the flow channel, so that the liquid flow in the flow channel becomes more stable. On the other hand, excessive flow rates increase the methanol crossover, resulting in cell performance degradation. Wei et al. [12] prepared a direct methanol fuel cell and investigated the effects of temperature, cathode humidification temperature, methanol concentration, methanol flow rate, and air flow rate on the polarization curve of the direct methanol fuel cell. The results showed that the best working condition of the cell was a temperature of 80 °C. In fact, the low current density working region uses a lower concentration of the methanol solution, whereas a high concentration methanol solution is supplied to the cell in the high current density region. For an active methanol fuel cell, the actual concentration of the methanol solution reacted in the catalytic layer can be adjusted by changing the flow rate of the methanol fuel at the inlet. The traditional variable speed liquid supply generally uses a microprocessor to monitor the current output, and then it adjusts the operating frequency of the micro pump according to the current feedback signal, thereby realizing real-time control of the flow rate. Although this method obviously increases the size and power consumption of the system, it is detrimental to the μDMFC’s portable applications.

Based on above understanding, in this paper, we developed a novel self-adaptive speed supply method derived from the internal reaction, where the design concept considers that the heat dissipated is different when the DMFC is in different working states, and thus the fuel supply speed in the microvalve is automatically adjusted. A microvalve was designed at the back side of the cell anode flow channel. For the methanol solution, the supplied flow rate is influenced by the dynamic viscosity related to the temperature of the solution. By changing the temperature of the methanol solution, the viscosity can be altered, and the variable speed can finally be achieved. Under low current density working conditions, the temperature of the methanol solution in the microvalve is low, and the micro valve outlet flow rate, that is, the inlet flow rate of the cell is also low, and this can meet the slower reaction requirements of the cell. When the reaction in the cell is gradually intensified with the increase in the working current, the increased heat generation is then transferred to the methanol solution inside the microvalve, and the outlet flow rate is gradually increased to satisfy the severe chemical reaction. Therefore, the microvalve structure can achieve an adaptive speed liquid supply mode, which effectively increases the power and stability during the entire process of the μDMFC operation. To further illustrate the method, this paper built a three-dimensional full-cell structure diagram with micro-valve using the COMSOL simulation software. The first part involved simulating the single micro-valve and verifying the relationship between viscosity and the speed of solution. The full cell model was simulated to illustrate the effect of the solution flow rate on cell performance, and the role of the microvalve structure. In addition to the simulation, we conducted experimental verifications, and the experimental results showed that the micro-valve could effectively improve the cell performance.

## 2. Simulation Analysis

**Figure 1:**
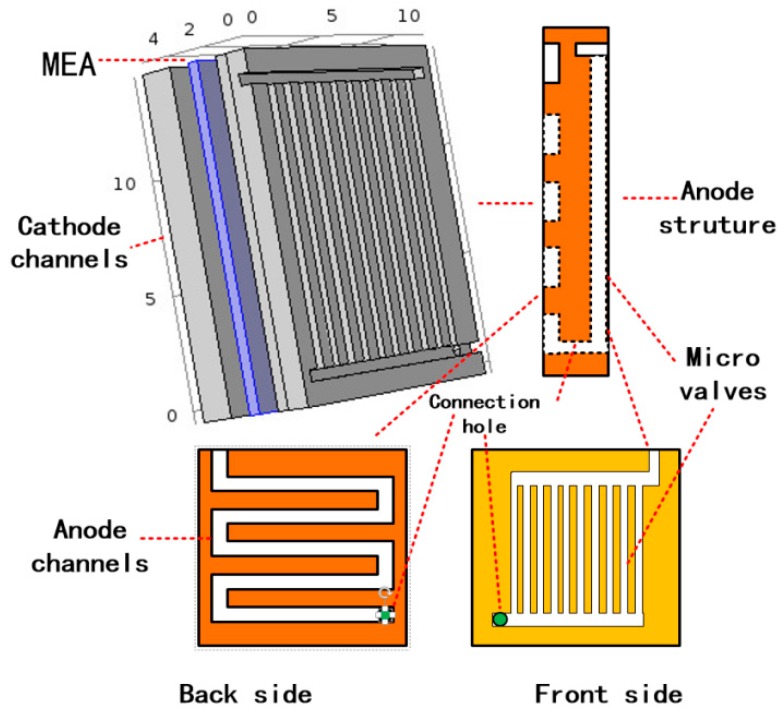
Three-dimension full cell model.

Figure 1 shows the three-dimensional full cell model with the microvalve. While studying the methanol solution state in the microvalve, to facilitate the calculation, a representative single channel was simulated with a cross-sectional area of 0.4 mm × 0.4 mm and a height of 10 mm. To study the mass transfer analysis and power density inside the μDMFC, we modeled the full cell calculation domain.

To simplify the calculation and model processing, some assumptions were made with regards to the mathematical model.

(1) The cell operates under stable state, single phase, and isothermal conditions.

(2) Since methanol reacts rapidly on cathode catalyst layer (CCL), it is assumed that the methanol reacts completely with anodic permeation.

(3) The electrochemical reaction is completed when only water and CO_2_ are produced, without any other side effects.

(4) The temperature of the outer wall of the cell is the same as that of the environment, ignoring the Joule heat generated by the internal resistance of the cell.

(5) Contact resistances between each layer are ignored.

(6) There are no gas fluxes through the membrane.

The fluid in the microvalve single-channel and anode channels was considered as in-compressible flow Newtonian fluids, and the single-phase laminar flow was used to describe the velocity field and pressure field in simulation. Therefore, the Navier–Stokes equation can be used to describe the momentum transfer, which is expressed as follows:

(1) $$\rho_{l}\left(u_{l}\cdot \nabla \right)u_{l}=-\nabla P_{ca}+\nabla \cdot \left[\mu_{l}\left(\nabla u_{l}+{\left(\nabla u\right)}^{T}\right)\right]$$

(2) $$\rho_{l}\left(\nabla \cdot u_{l}\right)=0$$

where $\rho_{l}$ represents the density of the fluid, $u_{l}$ is the liquid phase transport velocity in the flow channel, $P_{ca}$ is the pressure of the flow channel, $\mu_{l}$ is the dynamic viscosity coefficient of the fluid, and *T* denotes the temperature. Similarly, the momentum of oxygen in the cathode flow channel can be described using the above equation.

In the anode channels, owing to the diffusion and convection effect, the methanol was transported into the anode diffusion layer. The transport equation in the channel is given as:

(3) $$\nabla \cdot \left(-D_{m}\nabla c_{m}\right)+u_{l}\cdot \nabla c_{m}=0$$

Both the anode diffusion layer and the cathode diffusion layer are composed of a porous carbon cloth, so the Darcy law can be used to describe the momentum transfer in the porous medium, which can be expressed as follows:

(4) $$u_{l}=-\frac{\kappa }{\mu_{l}}\nabla P_{a}$$

where $u_{l}$ indicates the liquid phase transport velocity in the diffusion layer, *κ* represents the absolute permeability of the diffusion layer, and *P* represents the fluid pressure in the diffusion layer.

The mass transfer equation of liquid methanol in the anode diffusion layer is given by:

(5) $$\nabla \cdot \left(-D_{m}^{eff}\nabla c_{m}\right)+u_{l}\cdot \nabla c_{m}=0$$

The mass transfer equation of O_2_ in the cathode diffusion layer is given by:

(6) $$\nabla \cdot \left(-D_{O_{2}}^{eff}\nabla C_{O_{2}}\right)+u_{g}\cdot \nabla C_{O_{2}}=0$$

The mass transfer phenomenon in the PEM includes methanol crossover and water penetration. Methanol mass transfer consists of diffusion, convection, and elector-osmosis, where the total molar flux can be simplified as shown in this model:

(7) $$\nabla \cdot (-D_{m}^{mem,eff}\nabla c_{m})=0$$

(8) $$N_{cross}=-D_{m}^{mem,eff}\nabla c_{m}$$

where $D_{m}^{mem,eff}$ indicates the effective diffusion coefficient of methanol in the PEM, $c_{m}$ indicates the concentration of the methanol solution in the PEM, $N_{cross}$ is the flux of the methanol crossover.

Both the anode oxidation reaction and the reduction reaction of the cathode in the model are obtained by concentration dependent kinetics.

The current density expressions of the anode and cathode catalytic layer are shown as:

(9) $$i_{a}=i_{m}^{ref}\frac{c_{m}}{C_{m}^{ref}}\exp\left(\frac{\alpha_{a}F\eta_{a}}{RT}\right)$$

(10) $$i_{c}=i_{O_{2}}^{ref}\frac{C_{O_{2}}}{C_{O_{2}}^{ref}}\exp\left(-\frac{\alpha_{c}F\eta_{c}}{RT}\right)$$

where $i_{m}^{ref}$ is the anode reference exchange current density, $c_{m}$ is expressed as the concentration of the methanol solution on the anode catalyst layer, $C_{m}^{ref}$ represents the reference concentration of methanol, $\alpha_{a}$ represents the transfer coefficient of the anode catalyst layer, $i_{O_{2}}^{ref}$ represents the cathode reference exchange current density, and $C_{O_{2}}$ represents the oxygen concentration on the cathode catalyst layer.$C_{O_{2}}^{ref}$ indicates the reference concentration of oxygen, $\alpha_{c}$ represents the transfer coefficient of the cathode catalyst layer, whilst $\eta_{a}$ and $\eta_{c}$ are the over potential of the anode and cathode, respectively, which can be described as:

(11) $$\eta_{a}=\phi_{s}-\phi_{m}-E_{eq}^{a}$$

(12) $$\eta_{c}=\phi_{s}-\phi_{m}-E_{eq}^{c}$$

where $\phi_{s}$ is the electron potential and $\phi_{m}$ is the proton potential, $E_{eq}^{a}$ is the anode equilibrium voltage, and $E_{eq}^{c}$ is the cathode equilibrium voltage.

To obtain the effect of methanol crossover on the cathode overpotential, we assumed that the methanol permeated from the anode completed an electrochemical reaction on the cathode catalyst layer, and that the internal current $i_{p}$ could be described as:

(13) $$i_{p}=6FN_{cross}=i_{c}-i_{a}$$

The viscosity of the liquid was mainly caused by the cohesive force between the molecules. As the temperature increased, the molecular thermal motion increased, and the inter molecular cohesion weakened, causing the decrease in liquid viscosity. The estimated viscosity of the methanol aqueous solution is as described in Reference [13]:

(14) $$\ln\mu_{m}=x_{1}\ln\mu_{1}+x_{2}\ln\mu_{2}+ax_{1}x_{2}+bx_{1}^{2}x_{2}\ln x_{2}\left[1-\exp(-50x_{2}\right)]$$

where $\mu_{m}$ is the viscosity of the aqueous methanol solution, $x_{1}$ and $x_{2}$ are the molar fractions of component 1 (water) and component 2 (solute), respectively. $\mu_{1}$ and $\mu_{2}$ are the viscosities of component 1 (water) and component 2 (solute), respectively. The parameters a and b are the binary interaction parameters, which are related to the species type and temperature, and can be expressed by a linear relationship of temperature, as shown in Equations (11) and (12):

(15) $$a=a_{0}+a_{1}T$$

(16) $$b=b_{0}+b_{1}T$$

where $a_{0}=$ 5.37690, $a_{1}=$ −0.0115010, $b_{0}=$ −10.2113, $b_{1}=$ 0.0286300.

Heat transfer is a key process in the μDMFC, where we consider the heat generated by the electrochemical reaction. The heat flux generated by the oxidation of methanol on the anode catalyst layer can be expressed as:

(17) $$Q_{ACL}=i_{a}\eta_{a}-i_{a}\left(\frac{\Delta H_{a}-\Delta G_{a}}{6F}\right)$$

where $\Delta H_{a}$ is the enthalpy change corresponding to the oxidation of methanol, and $\Delta G_{a}$ represents the Gibbs free energy corresponding to the oxidation of methanol. In the above Equation, the first term on the right hand side represents the heat generated by the anode overpotential; whilst the second term represents the change in entropy corresponding to the anode methanol oxidation reaction.

Similarly, the heat flux corresponding to the reduction of oxygen on the cathode catalyst layer is shown as:

(18) $$Q_{CCL}=i_{c}\eta_{c}-i_{c}\left(\frac{\Delta H_{c}-\Delta G_{c}}{4F}\right)-i_{p}\left(\frac{\Delta H_{a}-\Delta G_{a}}{6F}\right)$$

where, the first term on the right side of the Equation represents the mixed potential caused by methanol permeation and the heat generated by the cathode overpotential; whilst the second term represents the change in entropy corresponding to the oxygen reduction reaction; and the third term indicates the entropy change of oxygen and methanol which permeate into the cathode catalyst layer.

For the microvalve’s anode flow path and the cathode flow path, only the fluid heat transfer in the flow path needs to be considered. Therefore, the heat transfer equations in the anode and cathode channels can be expressed as follows:

(19) $$\rho C_{p}u\cdot \nabla T-\nabla \cdot \left(k\nabla T\right)=Q$$

where $C_{p}$ is the specific heat capacity of the fluid, and *k* is expressed as the heat transfer coefficient.

For the anode and cathode diffusion layers, these are composed of porous media. Therefore, the heat transfer in the anode and cathode diffusion layer is shown as:

(20) $$\rho C_{p}u\cdot \nabla T-\nabla \left(k^{eff}\nabla T\right)=Q$$

The PEM is a solid perfluoro sulfonic acid type membrane, such that heat transfer therein can be considered to be transported in solids. The anode and cathode plates also experience heat transfer in solids. Therefore, the heat transfers in the PEM and the two plates can be expressed as follows, respectively:

(21) $$-\nabla \cdot \left(k_{mem}\nabla T\right)=Q$$

(22) $$-\nabla \cdot \left(k_{cc}\nabla T\right)=Q$$

where $k_{cc}$ indicates the thermal conductivity of the plate, and $k_{mem}$ is the thermal conductivity of the PEM.

In this study, the finite element analysis solver, COMSOL Multiphysics, was used to develop the above model. After constructing the above equations into the model, parameters and variables are added to the equation for physical domain setting, boundary setting, meshing, and solving. Table 1 describes some of the parameters and variables used in the model solution.

## 3. Results and Discussion

### 3.1. Adaptive-Speed Microvalve Design

The heat generated by the reaction was transferred to the flow channel to cause a change in the physical properties of the methanol solution, particularly the viscosity of the solution. There are two reasons for the viscosity of fluids: the cohesive force between molecules, and the exchange of momentum generated by the thermal motion between molecules. The viscosity of the liquid was mainly caused by the cohesive force between molecules. As the temperature increased, the molecular thermal motion increased, and the inter molecular cohesion weakened, causing the decrease in viscosity.

**Figure 2:**
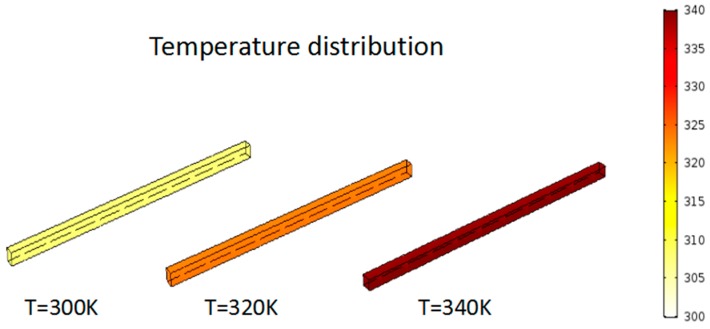
Temperature distribution in the microvalve under different heating conditions.

**Figure 3:**
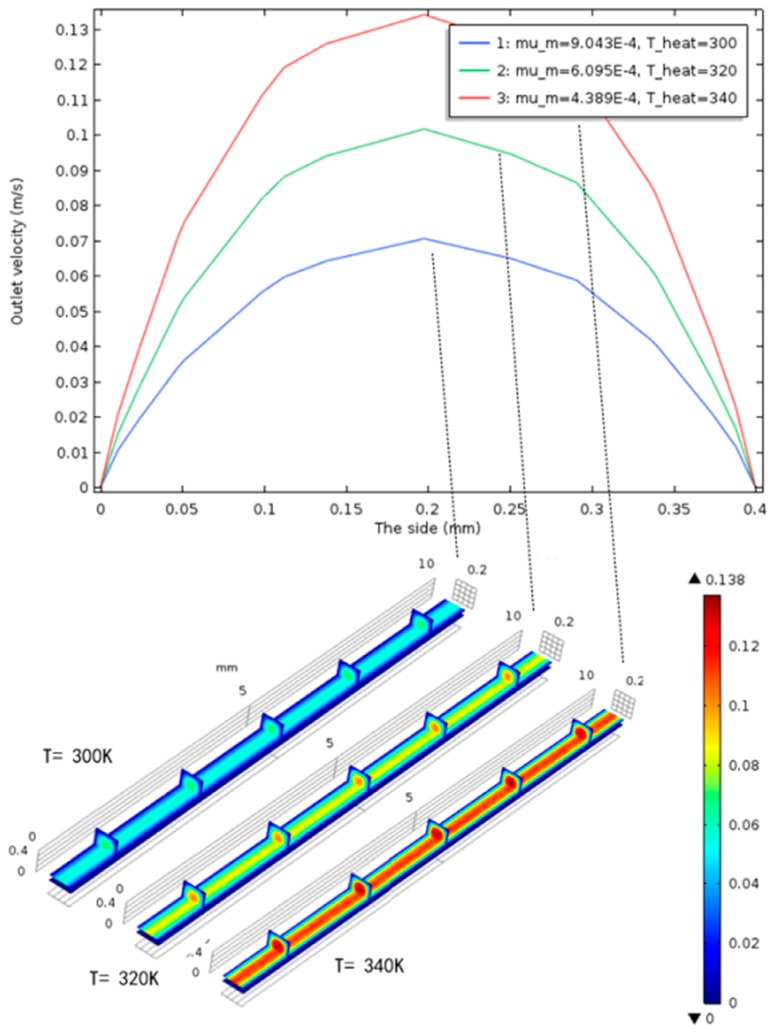
Velocity distribution in the microvalve under different heating conditions.

**Figure 4:**
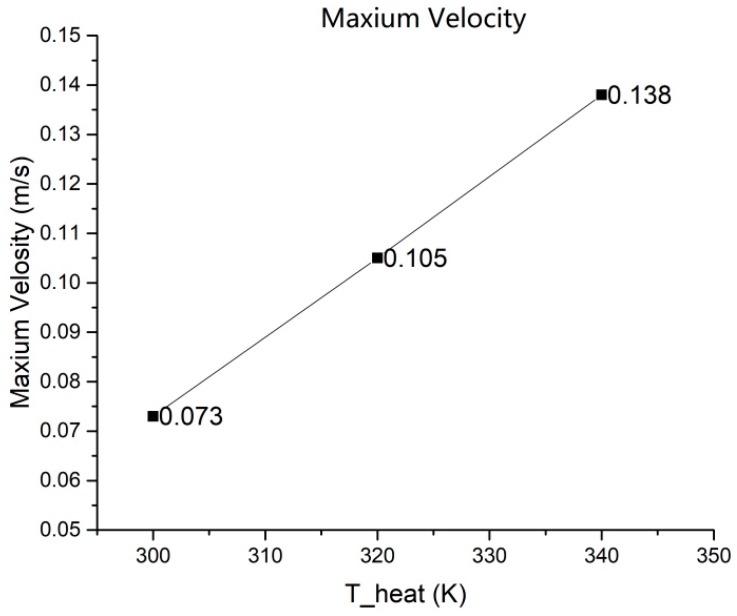
Maximum outlet velocity variation with heat temperatures.

It should be noted that, in Figure 1, it can be seen that the structure of the microvalve consists of two parts, where the microvalve structure is the narrower middle channel and the other parts are the upper and lower two channels, which are responsible for connecting the middle channels. First, the solution flows through the upper channel to the middle microvalve channel, and then into the anode channel at the bottom channel. Therefore, the structures of the middle microvalve are the same size with each other. We can use the simulation of the single microvalve structure to illustrate the problem. In the model simulation, the side of the microvalve that was in contact with the cell was heated, and the temperature, dynamic viscosity, and velocity distribution of the methanol solution in the microvalve were observed by changing the heating temperature from 300 K to 340 K. The results showed that as the heating temperature rose from 300 K to 340 K, there was a change in the calculations of the viscosity of the methanol solution, as shown in Table 2. The model simulation shows that the temperature distribution in the microvalve is uniform under different heat conditions, as shown in Figure 2, whilst the corresponding velocity distribution is shown in Figure 3. The velocity distribution in the microvalve was uneven due to the influence of wall resistance, and also the velocity in the middle position was faster. Figure 4 shows the velocity at the mid-line of the microvalve outlet section, where the maximum value of the solution flow velocity at the outlet of the channel increased by 90%.

**Figure 5:**
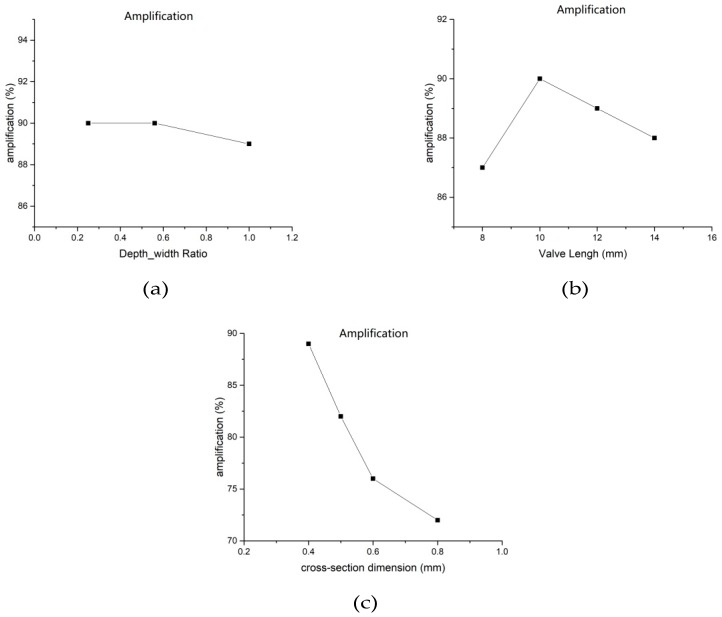
Effect of the microvalve geometry change on the outlet flow rate: (**a**) Depth-width Ratio (**b**) Valve length (**c**) Cross-section dimension.

We also simulated the microvalve geometry to highlight the effect of the control speed change of the microvalve under changes in temperature. We observed a change in the flow rate of the methanol solution outlet, as the temperature in the microvalve was changed from 300 K to 340 K. Figure 5 shows the effect of the depth–width ratio, cross-section dimension, and valve length of the microvalve channel, respectively. Figure 5 shows that the depth–width ratio of the microvalve channel had little effect on the increase in the outlet flow velocity, and thus the outlet cross-section ratio of 1:1 was selected (refer to our previous research results). By adjusting the cross-section dimension and the length of the microvalve, the results from Figure 5 indicate that the cross-section has a great influence on the increase in outlet flow velocity. In combination with the overall geometry of the μDMFC, a cross-sectional area of 0.4 mm × 0.4 mm and a length of 10 mm were preferred.

### 3.2. Effect of Adaptive Flow Rate on Cell Output Performance

Previous studies have shown that a flow rate of 1 mL/min is the optimal flow rate for the μDMFC. However, this conclusion was only obtained by comparing the maximum output power. Considering the special conditions in the μDMFC operation process, the simulation of the full cell is divided into two main regions, namely a small current density area and a large current density area. Different from the constant flow conditions of the methanol solution in the previous study, the different flow rates corresponding to the two-part working range were exhibited. It could be clearly seen from the model that the microvalve outlet speeds were 0.073 m/s (0.7 mL/min) and 0.138 m/s (1.3 mL/min) under heating conditions of 300 K and 340 K, respectively.

**Figure 6:**
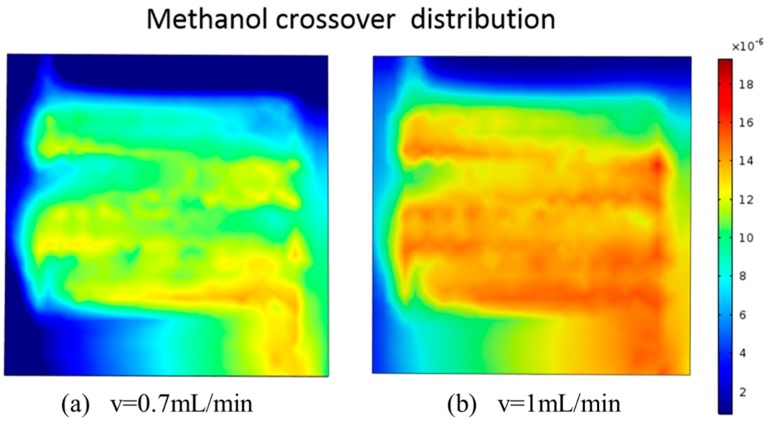
The methanol permeability at inlet methanol velocity is: (**a**) 0.7 mL/min and (**b**) 1 mL/min.

The anode reactant methanol solution permeates to the cathode through the PEM, and a mixed potential is generated when the surface of the cathode catalyst layer reacts with the oxidant oxygen, thereby causing a drop in the overall voltage of the cell. This phenomenon is also known as the methanol crossover. When studying the cell at a small current density, it is assumed that the operating temperature is 300 K, and Figure 6 shows the amount of methanol crossover at the cathode diffusion layer at inlet methanol flow rates of 0.7 mL/min and 1 mL/min, respectively. We observed that at a flow rate of 0.7 mL/min methanol solution, the methanol permeation amount was significantly lower than that at the working condition of 1 mL/min. At this time, the low-flow methanol solution could satisfy the normal chemical reaction requirements of the cell. Excessive methanol solution will result in a severe methanol crossover, which will lead to a reduction in the total voltage.

**Figure 7:**
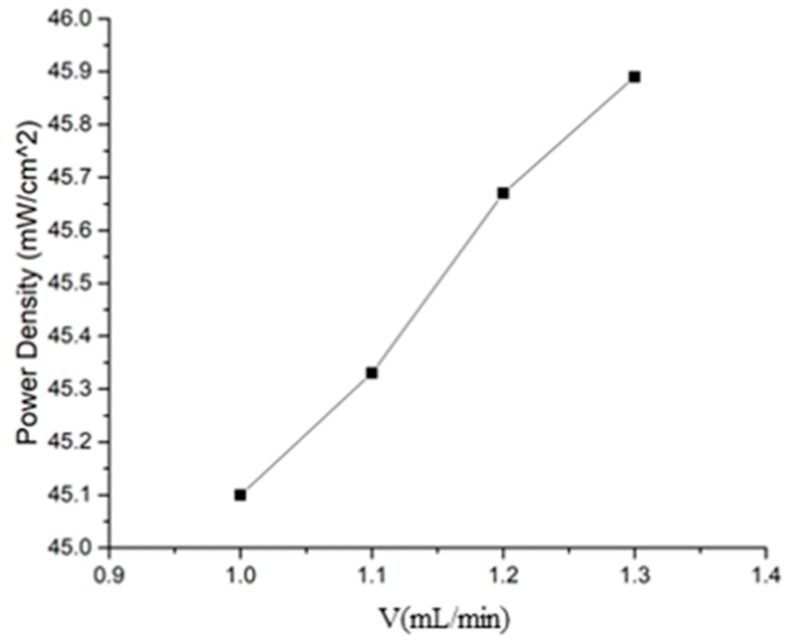
The output power variation with the inlet methanol velocity.

When studying cells at high current densities, it is assumed that the μDMFC has been in operation for a period of time, and that the operating temperature is 340 K. The catalyst is more effective, requiring more methanol fuel to satisfy the violent chemical reaction. At this working stage, increasing the methanol flow rate at the inlet can effectively increase the amount of methanol reactants involved in the reaction. As shown in Figure 7, the output power density increases as the inlet flow rate increases from 1 mL/min to 1.3 mL/min.

### 3.3. Experimental Performance Comparison between the Adaptive Cell and Conventional Cell

**Figure 8:**
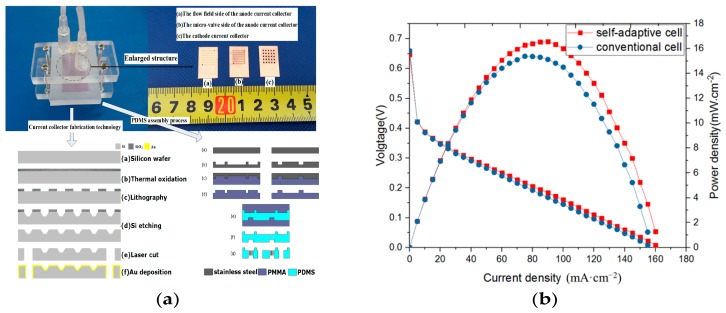
The micro DMFC and the performances between the cell with a microvalve and a conventional cell. (**a**) The cell fabrication structure, (**b**) I-V and I-P curve.

To detect the whole process of cell operation, the self-adaptive micro DMFC based on the microvalve, as well as a conventional cell, were fabricated and assembled, wherein the effective areas were both 1 cm × 1 cm, as shown in Figure 8. The self-adaptive cell and the conventional cell were injected with 2 mol/L of methanol solution with an inlet flow rate of 0.7 mL/min and 1 mL/min, respectively. During the experiment, we controlled the only variable being the methanol inlet flow rate, and the rest of the conditions, including methanol concentration, temperature, and pressure, were all consistent. This ensured that the difference in the experimental results would be due to the different entrance methanol velocities, where the test results are shown in Figure 8. The optimum performance of the self-adaptive cell was 16.56 mW/cm^2^, which was obtained at a 90 mA/cm^2^ current density. The optimum performance of the conventional cell was obtained at a 75 mW/cm^2^ current density, with a maximum power density of 15.37 mW/cm^2^. The improved performance was attributed to the self-adaptive cell being able to achieve a more adequate methanol reactant at higher current densities.

## 4. Conclusions

In this paper, we designed a microvalve speed control structure to achieve a self-adaptive fuel supply mechanism, based on the relationship between the viscosity, velocity, and temperature of the methanol solution at different operating current densities. Through the simulation of a microvalve single-flow channel model, we obtained the velocity distribution at different temperatures and different viscosities. In the process of changing the temperature from 300 K to 340 K, the microvalve outlet speed was altered by nearly 90%. Through the model calculation of the whole μDMFC, the results showed that the cell operating at a small current density effectively suppressed the methanol permeation. When the current density increased, the heat generated by the cell increased, and the flow rate at the outlet of the microvalve increased from 1 mL/min to 1.3 mL/min, which could effectively increase the power density. We further verified from an experimental comparison between the self-adaptive cell (16.56 mW/cm^2^) and the conventional cell (15.37 mW/cm^2^) that the output power of the μDMFC had improved. Therefore, the microvalve structure allows the cell to adaptively adjust the flow rate of the methanol solution without external equipment, satisfying the proper demand for reactants in the whole working process, and effectively improving the working efficiency and stability. The proposed design can provide some motivation for portable integrated applications of micro direct methanol fuel cells.

## Figures and Tables

**Table 1:** Variables and parameters in the model.

Descriptions	Parameters	Values
Pressure constant	P	1 [atm]
Density, H_2_O	$\rho_{H_{2}O}$	1000 [kg/m^3^]
Density, Methanol	$\rho_{m}$	791.7 [kg/m^3^]
Viscosity, Gas phase	$\mu_{g}$	14.96 × 10^−6^ [kg/(m·s)]
Permeability, GDL		1 × 10^−12^ [m^2^]
Porosity, AGDL CGDL	$\varepsilon_{d}$	0.7
Electric conductivity, AGDL CGDL	$\sigma_{s}$	4000 [S/m]
Reference concentration, Methanol	$c_{m}$	1000 [mol/m^3^]
Reference concentration, O_2_	$c_{o_{2}}$	0.21P/(R·T) [mol/m^3^]
Transfer coefficient, Anode	$\alpha_{a}$	0.239
Transfer coefficient, Cathode	$\alpha_{c}$	0.875
Liquid methanol enthalpy	$\Delta H_{m}$	−238.66 × 10^3^ [J/mol]
Liquid water enthalpy	$\Delta H_{h_{2}o}$	−285.83 × 10^3^ [J/mol]
Carbon dioxide enthalpy	$\Delta H_{co_{2}}$	−393.51 × 10^3^ [J/mol]
Liquid methanol Gibbs free energy	$\Delta G_{m}$	−166.27 × 10^3^ [J/mol]
Liquid water Gibbs free energy	$\Delta G_{h_{2}o}$	−237.08 × 10^3^ [J/mol]
Carbon dioxide Gibbs free energy	$\Delta G_{co_{2}}$	−394 × 10^3^ [J/mol]
Gas constant	R	8.314 [J/(mol.K)]
Faraday’s constant	F	96485 [C/mol]
Reference concentration, O_2_	$c_{o_{2}}$	0.21 P/(R.T)
Liquid methanol specific heat capacity	$C_{pm}$	2530 [J/(kg·K)]
Gas phase specific heat capacity	$C_{pg}$	1005 [J/(K·kg)]
Thermal conductivity, Plate	$k_{cc}$	50 [W/(K·m)]
Thermal conductivity, Membrane	$k_{mem}$	0.21 [W/(K·m)]
Thermal conductivity, GDL	$k_{gdl}$	1.7 [W/(K·m)]
Thermal conductivity, Methanol	$k_{m}$	0.21 [W/(K·m)]
Thermal conductivity, Water	$k_{h_{2}o}$	0.58 [W/(K·m)]
Thermal conductivity, Gas	$k_{g}$	0.0257 [W/(K·m)]
Diffusion coefficient, Methanol	$D_{m}$	2.8 × 10^−9^ exp(2436 × (1/333 − 1/T)) [m^2^/s]
Diffusion coefficient, O_2_	$D_{o_{2}}$	1.775 × 10^−5^ × (T/273)1.823 [m^2^/s]
Diffusion coefficient in PEM, methanol	$D_{m}^{mem,eff}$	4.9 × 10^−10^ exp(2436 × (1/333 − 1/T)) [m^2^/s]
Anode equilibrium potential	$E_{eq}^{a}$	−1 × (131350 − 408.22 × T)/(6 × F) [V]
Cathode equilibrium potential	$E_{eq}^{c}$	−1 × (−285830 × 3 + 489.52 × T)/(6 × F) [V]
Anode referential current	$i_{m}^{ref}$	94.25 exp(35570 × (1/353 − 1/T)) [A/m^2^]
Cathode referential current	$i_{O_{2}}^{ref}$	0.04222 exp(73200/R × (1/353 − 1/T)) [A/m^2^]

**Table 2:** Methanol solution viscosity at different temperatures.

Temperature (K)	Viscosity (Pa·S)
300	0.0009043
320	0.0006095
340	0.0004389

## Nomenclature

μDMFC	micro direct methanol fuel cell
CCL	cathode diffusion layer
PEM	proton exchange membrane
AGDL	anode gas diffusion layer
CGDL	cathode gas diffusion layer
